# Development of peak oxygen uptake from 11–16 years determined using both treadmill and cycle ergometry

**DOI:** 10.1007/s00421-019-04071-3

**Published:** 2019-01-09

**Authors:** Neil Armstrong, Jo Welsman

**Affiliations:** Children’s Health and Exercise Research Centre, University of Exeter, St Lukes Campus, Heavitree Road, Exeter, EX1 2LU UK

**Keywords:** Aerobic fitness, Children, Health-related cut-points, Ergometry, Fat-free mass, Multilevel modelling

## Abstract

**Purposes:**

To investigate the development of peak oxygen uptake ($\dot{{V}}{\text{O}}_{2}$) assessed on both a treadmill and a cycle ergometer in relation with sex and concurrent changes in age, body mass, fat-free mass (FFM), and maturity status and to evaluate currently proposed ‘clinical red flags’ or health-related cut-points for peak $\dot{{V}}{\text{O}}_{2}$.

**Methods:**

Multiplicative multilevel modelling, which enables the effects of variables to be partitioned concurrently within an allometric framework, was used to analyze the peak $\dot{{V}}{\text{O}}_{2}$s of 138 (72 boys) students initially aged 11–14 years and tested on three annual occasions. Models were founded on 640 (340 from boys) determinations of peak $\dot{{V}}{\text{O}}_{2}$, supported by anthropometric measures and maturity status.

**Results:**

Mean peak $\dot{{V}}{\text{O}}_{2}$s were 11–14% higher on a treadmill. The data did not meet the statistical assumptions underpinning ratio scaling of peak $\dot{{V}}{\text{O}}_{2}$ with body mass. With body mass appropriately controlled for boys’ peak $\dot{{V}}{\text{O}}_{2}$s were higher than girls’ values and the difference increased with age. The development of peak $\dot{{V}}{\text{O}}_{2}$ was sex-specific, but within sex models were similar on both ergometers with FFM the dominant anthropometric factor.

**Conclusions:**

Data should not be pooled for analysis but data from either ergometer can be used independently to interpret the development of peak $\dot{{V}}{\text{O}}_{2}$ in youth. On both ergometers and in both sexes, FFM is the most powerful morphological influence on the development of peak $\dot{{V}}{\text{O}}_{2}$. ‘Clinical red flags’ or health-related cut-points proposed without consideration of exercise mode and founded on peak $\dot{{V}}{\text{O}}_{2}$ in ratio with body mass are fallacious.

## Introduction

Peak oxygen uptake (peak $\dot{{V}}{\text{O}}_{2}$) is internationally recognized; as the criterion measure of youth aerobic fitness and in paediatric exercise laboratories, it is routinely determined either running on a treadmill or pedaling on a cycle ergometer (Falk and Dotan 2018; McManus and Armstrong 2017). Current understanding of the development of aerobic fitness in youth is based on an amalgam of data from these ergometers, but the influence of mode of exercise in relation to sex-specific, concurrent changes in body mass, fat-free mass (FFM), and maturity status on peak $\dot{{V}}{\text{O}}_{2}$ is unexplored.

Cross-sectional comparisons of boys aged 10–14 years have reported cycle ergometer- and treadmill-determined values of peak $\dot{{V}}{\text{O}}_{2}$ to be highly correlated but treadmill-determined values have consistently been noted as 7–15% higher than those achieved on a cycle ergometer (Boileau et al. 1977; Duncan et al. 1996; Macek et al. 1976). This is probably due to the greater muscle mass, enhanced venous return, increased stroke volume (SV), and reduced peripheral resistance during treadmill running. Peak $\dot{{V}}{\text{O}}_{2}$ when running on a treadmill is therefore more likely to be limited by cardiovascular factors than peripheral factors such as local muscle fatigue. Despite well-documented sex differences in the development of peak $\dot{{V}}{\text{O}}_{2,}$ there are no comparative data on girls. Yet, regularly cited reviews have interpreted the extant literature on the development and training of youth aerobic fitness in both sexes by pooling treadmill- and cycle ergometer-determined peak $\dot{{V}}{\text{O}}_{2}$ values (e.g., Bacquet et al. 2003; Bar-Or and Rowland 2004; Pfeiffer et al. 2008). Other authors have ‘corrected’ for ergometer differences by multiplying cycle ergometer values by different fixed percentages and pooling data regardless of sex, age, or maturity status (e.g., Aadland et al. 2018; Krahenbuhl et al. 1985; Stavnsbo et al. 2018).

There is a plethora of both treadmill- and cycle ergometer-based cross-sectional studies of youth aerobic fitness with the vast majority attempting to control for growth by focusing on a single anthropometric variable and interpreting peak $\dot{\text{V}}{\text{O}}_{2}$ in ratio with body mass (see Armstrong and Welsman 1994 for review). Longitudinal studies are sparse with some founded on cycle ergometer determinations of peak $\dot{{V}}{\text{O}}_{2}$ (e.g., Cunningham et al. 1994; Janz et al. 1998; Rutenfranz et al. 1981) and others on treadmill determinations (e.g., Mirwald and Bailey 1986; Rowland et al. 1997; Sprynarova et al. 1987), but no longitudinal studies have investigated performance on both ergometers. Data analysis generally consists of a series of annual cross-sectional examinations of peak $\dot{{V}}{\text{O}}_{2}$ (i.e. in L·min^− 1^) and peak $\dot{{V}}{\text{O}}_{2}$ ratio-scaled with body mass (i.e., in mL·kg^− 1^·min^− 1^). Collectively longitudinal studies indicate boys’ peak $\dot{{V}}{\text{O}}_{2}$ to increase in a near-linear manner from 11 to 16 years with girls’ peak $\dot{\text{V}}{\text{O}}_{2,}$ showing a similar trend before levelling-off from ~ 13 years of age. Boys’ peak $\dot{{V}}{\text{O}}_{2}$ ratio-scaled with body mass has been reported to remain unchanged from 11 to 16 years, while girls’ values decrease with age, particularly from ~ 13 years of age (see Armstrong and McManus 2017 for review). The experimental designs, statistical analyses, and data interpretation in the extant literature have, however, revealed limited insights into the development of youth aerobic fitness which is influenced by concurrent changes in several variables.

Allometric scaling has challenged the ‘convenient and traditional’ (Bar-Or and Rowland 2004) interpretation of data and demonstrated, in contrast with ratio scaling, that with body mass appropriately controlled for there is a progressive increase in youth peak $\dot{{V}}{\text{O}}_{2}$ with age in both sexes (Welsman et al. 1996). However, it is the emergence (Aitkin et al. 1981) and regular refinement (Rasbash et al. 2018) of multilevel regression modelling which has opened up new analytical approaches to developmental exercise physiology. Multilevel modelling enables the effects of variables such as age, body mass, FFM, and maturity status to be partitioned concurrently within an allometric framework to provide a flexible and sensitive interpretation of exercise performance variables. In contrast to traditional methods that require a complete longitudinal data set, both the number of observations per individual and the temporal spacing of the observations may vary within a multilevel model. In an innovative re-analysis of previously published data of elite youth athletes Nevill et al. (1998) introduced multiplicative, allometric modelling to paediatric sport science and with the present authors applied it to interpreting growth and maturation changes in peak oxygen uptake from 11–13 years (Armstrong et al. 1999). To date, no study has used this technique to analyze the development of youth peak $\dot{{V}}{\text{O}}_{2}$ determined concomitantly on a treadmill and a cycle ergometer.

As fat mass does not make a significant contribution to peak $\dot{{V}}{\text{O}}_{2}$ (Goran et al. 2000) FFM is likely to be a more relevant morphological variable than body mass in the development of aerobic fitness. A case can be made for determining FFM on each test occasion, but likely due to ethical and/or resource limitations, no study has been published which includes several hundred serial determinations of peak $\dot{{V}}{\text{O}}_{2}$ and FFM. Moreover, measures of body fat of the same young people have been shown to vary widely across established laboratory techniques (Ferri-Morales et al. 2018). In the few studies in which FFM has been reported estimates from body mass and skinfold thicknesses have provided a pragmatic morphological variable with which to study longitudinal changes in peak $\dot{{V}}{\text{O}}_{2}$ (e.g., Cunningham et al. 1994; Janz et al. 1998; Rowland et al. 1997). FFM is typically predicted from body mass and skinfold thicknesses using the youth-specific equations developed by Slaughter et al. (1988), but validation studies of the equations have revealed wide limits of agreement and a tendency to under-predict fat in girls and over-predict fat in boys (Roemmich et al. 1997). Multiplicative, allometric modelling offers the opportunity to consider body mass and skinfold thicknesses together as a surrogate for FFM. A recent longitudinal study of 11–18-year-old youth demonstrated that skinfold thicknesses and body mass together explained more of the variance in short-term power output than the estimation of FFM from youth-specific equations (Armstrong and Welsman 2018a). Prior to the present project, this approach had not been investigated in relation with aerobic fitness.

Given the recent surge in papers calling for the raising of ‘clinical red flags’ or the establishment of cut points of ‘cardiometabolic risk’ based on values of peak $\dot{{V}}{\text{O}}_{2}$ ratio-scaled with body mass (e.g., Agbaje et al. 2018; Lang et al. 2017; Ruiz et al. 2016), the need to clarify the effects of concurrent changes in morphological covariates and maturity status on sex-specific changes in youth aerobic fitness with age has become critically important. Ratio scaling does not have a rigorous scientific rationale, is seldom statistically justified, favours lighter individuals, but penalizes heavier youth and leads to spurious correlations with other health-related variables (Tanner 1949; Welsman and Armstrong 2018; Winter and Nevill 2009). For example, the statistical association of cardiovascular risk factors with the ratio-scaled peak $\dot{{V}}{\text{O}}_{2}$ of overweight/obese children is likely to reflect overweight/obese status to a greater extent than aerobic fitness (Loftin et al. 2016).

‘Clinical red flags’ and ‘cardiometabolic risk’ cut points have been proposed without reference to mode of exercise. Cycle ergometer peak $\dot{{V}}{\text{O}}_{2}$s have been ‘corrected’ to treadmill values by multiplying by 1.05 (Stavnsbo et al. 2018), based on a previous observation of a 5% difference in 20 9-year-old boys (Mamen et al. 2009). Another recent report promoted age-related ‘aerobic fitness thresholds’ to define poor metabolic health from 8–18 years extrapolated from cross-sectional cycle ergometer-determined peak $\dot{{V}}{\text{O}}_{2}$ values from 9 to 15 years and suggested that, ‘raising our cut-points by ~ 2–3 mL·kg^− 1^·min^− 1,^ would make them equivalent to values obtained by a treadmill protocol’ (Aadland et al. 2018). It is apparent that current ‘clinical red flags’ (and similar health-related peak $\dot{{V}}{\text{O}}_{2}$ cut-points) for the age range 8–18 years do not take appropriate account of mode of exercise, maturity status, morphological variables other than body mass (ratio-scaled with peak $\dot{{V}}{\text{O}}_{2}$), and, in some cases, age. They, therefore, have the potential to misinform clinical practice, mislead policy statements and misguide recommendations designed to promote youth health (Armstrong and Welsman 2018b) and require further scrutiny within a longitudinal framework across different modes of exercise.

To inform developmental exercise physiology and to contribute to a sound scientific foundation for health-related recommendations for youth, clarification of the influence of exercise mode and concurrent sex-specific changes in age, maturity status, and morphological covariates is required. The purposes of the present study were, therefore, (1) to adopt a multiplicative allometric approach to investigate the influence of mode of exercise on peak $\dot{{V}}{\text{O}}_{2}$ from 11–16 years in relation with sex and concurrent changes in age, body mass, FFM, and maturity status and (2) to evaluate the data in relation with current proposals of ratio-scaled ‘clinical red flags’ and similar health-related cut-points for youth peak $\dot{{V}}{\text{O}}_{2}$.

## Methods

### Participants

136 (72 boys) students aged 11–14 years volunteered to participate in a series of studies of aerobic and anaerobic fitness. Individual ages were computed from date of birth at each test session. The studies received ethical approval from the District Health Authority Ethical Committee and all participants and their guardians provided written informed consent to participate in the studies. The treadmill-determined data have been integrated into a review of over 1000 treadmill determinations of youth peak $\dot{{V}}{\text{O}}_{2}$ reported elsewhere (Armstrong and Welsman 2018c), but neither of the present data sets have previously been reported.

### Experimental procedures

Participants visited the Research Centre on three annual occasions and were well-habituated to the laboratory environment, to the laboratory personnel who were unchanged throughout the study, and to the experimental procedures. The annual exercise tests took place in randomized order with a 1 day rest interval between tests. The same equipment and procedures were used throughout the study. Body mass was assessed using Avery balance scales (Avery, Birmingham, UK), and skinfold thicknesses over the triceps and subscapular regions were measured using Holtain skinfold calipers (Holtain, Crmych, Dyfed, UK). Apparatus was calibrated according to the manufacturers’ instructions. Maturity status was visually assessed by the Research Centre nurse using the indices for pubic hair (PH) development described by Tanner (1962). FFM was estimated from skinfolds, body mass, and maturity status using youth-specific equations (Slaughter et al. 1988).

Following a standardized warm-up, peak $\dot{{V}}{\text{O}}_{2}$ was determined during progressive, incremental exercise tests to voluntary exhaustion on a motorized treadmill (Woodway, Cranlea Medical, Birmingham, UK) and an electronically braked cycle ergometer (Lode Excalibur Sport, Groningen, Netherlands). Heart rate (HR) was monitored with electrocardiography and expired gases were monitored continuously using an Oxycon Sigma on-line gas-analysis system (Cranlea Medical) which was calibrated prior to each test using gases of verified concentration. Depending on age, treadmill tests began at a belt speed of 1.94 m·s^− 1^ (7 km·h^− 1^) or 2.22 m·s^− 1^ (8 km·h^− 1^) and increased by 0.28 m·s^− 1^ (1 km·h^− 1^) every 2 min until a speed of 2.78 m·s^− 1^ (10 km·h^− 1^) was reached. Subsequently, belt speed was held constant and the gradient was incrementally increased by 2.5% every 2 min until voluntary exhaustion. Cycle ergometer tests commenced at an exercise intensity of either 60 or 80 W depending on age. The pedal cadence was fixed at 60 rpm and the exercise intensity increased by 20 W every 2 min until the children were exhausted and could no longer maintain the pedal cadence. The highest 30 s $\dot{{V}}{\text{O}}_{2}$ attained was accepted as a maximal index if clear signs of intense exertion (e.g., hyperpnea, facial flushing unsteady gait, and profuse sweating) were demonstrated and supported by a respiratory exchange ratio greater than 1.00 and a HR which was levelling-off over the final stages of the test at a value within 5% of the mean ergometer-specific maximal HRs we have previously reported for boys and girls aged 11–16 years (Armstrong et al. 1991). All participants reported in this study satisfied these criteria on all test occasions.

### Data analyses

Data were analysed using SPSS v25 (IBM SPSS Statistics). To describe age, body mass, and estimated FFM relationships with peak $\dot{{V}}{\text{O}}_{2}$, data were graphed by ergometer and sex and Pearson product moment correlation coefficients were computed. Mean age-related differences and individual variations between treadmill- and cycle ergometer-determined peak $\dot{{V}}{\text{O}}_{2}$s were determined by sex.

Factors associated with the development of treadmill- and cycle ergometer-determined peak $\dot{{V}}{\text{O}}_{2}$ were analysed using multilevel regression modelling (MLWin v3.02, Centre for Multilevel Modelling, University of Bristol, UK). The multiplicative, allometric approach introduced by Nevill et al. (1998) and implemented in subsequent analyses of youth exercise performance (e.g. Armstrong and Welsman 2018a, c; De Ste Croix et al. 2002) was adopted (Eq. 1):

1 $$y = {\rm{mas}}{{\rm{s}}^{{k_1}}}\cdot\exp {\rm{ }}({a_j} + b\cdot{\rm{age}}{\mkern 1mu} + {\mkern 1mu} c\cdot{\rm{ag}}{{\rm{e}}^2})eij.$$

Log transformation linearized the model as in Eq. 2 and this formed the starting point for analyses:

2 $${\log _e}y={k_1}\cdot{\log _e}{\text{mass}}+{a_j}+{b_j}\cdot{\text{age}}+c{\text{ }}\cdot{\text{ag}}{{\text{e}}^2}+{\log _e}(eij).$$

All parameters were fixed with the exception of the constant (*k*) (intercept term) which was allowed to vary randomly at level 2 (between individuals) and the multiplicative error ratio (*ε*) which also varied randomly at level 1 (within individual) as denoted by the subscripts *i* (level 1 variation) and *j* (level 2 variation). Age was centred on the group mean. From the baseline model of age, age^2^, and body mass, additional explanatory variables were explored. In the initial models sex differences were investigated using the indicator variable boys = 0, girls = 1 which sets the boys’ constant as the baseline from which the girls’ parameter is allowed to deviate. The interaction term age by sex investigates differential development of peak $\dot{\text{V}}{\text{O}}_{2}$ between girls and boys and the age^2^ term indicates changes in the size of the age effect as the rate of change in growth decreases. In the sex-specific analyses, from the baseline model of age, age^2^, and body mass (or estimated FFM), additional explanatory variables including indicator variables for maturity status (i.e. PH stages 2, 3, 4, and 5) which set stage 1 as the baseline from which effects due to maturation can be explored. With age and body mass (but not estimated FFM) models, sum of triceps and subscapular skinfold thicknesses was also entered.

Parameter estimates were considered significant (*p* < 0.05) when their value exceeded 2 × the standard error (SE). A comparison of the goodness of fit of the different models was obtained from the change in the deviance statistic (− 2 × log-likelihood) with reference to the number of fitted parameters. In a comparison of two models with the same number of fitted parameters, the model with the smallest − 2 × log-likelihood reflects that with the best fit. Additional parameters contribute to improved fit from the change in − 2 × log-likelihood according to a Chi-squared statistic for additional degrees of freedom added.

## Results

### Descriptive data

**Fig. 1:**
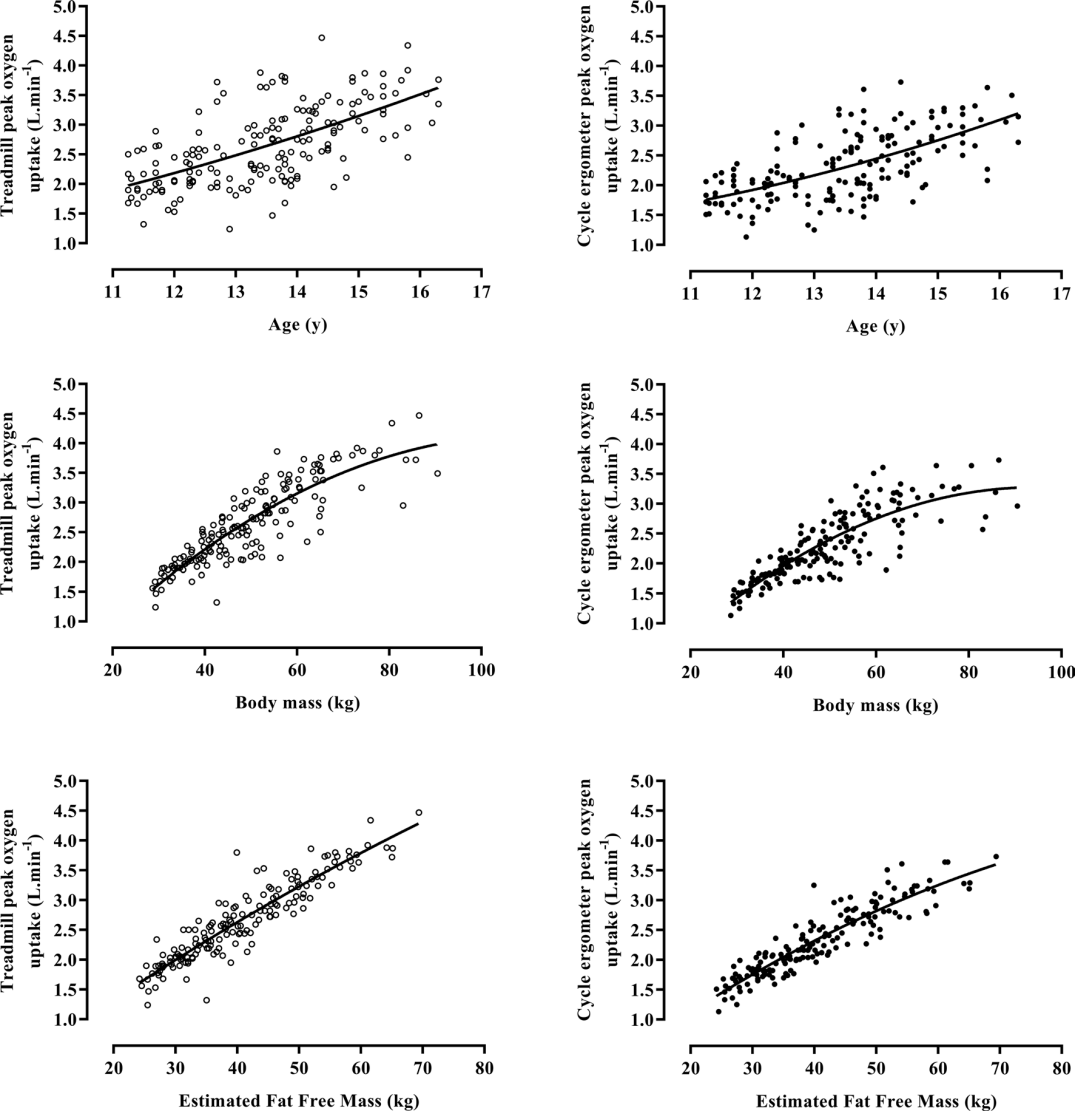
Treadmill and cycle ergometer-determined peak oxygen uptake by age, body mass, and fat-free mass in 11–16-year-old boys. Data are from 170 determinations of 11–16-year-old boys’ peak oxygen uptake on each ergometer. Fat-free mass is estimated from youth-specific equations (Slaughter et al. 1988)

**Fig. 2:**
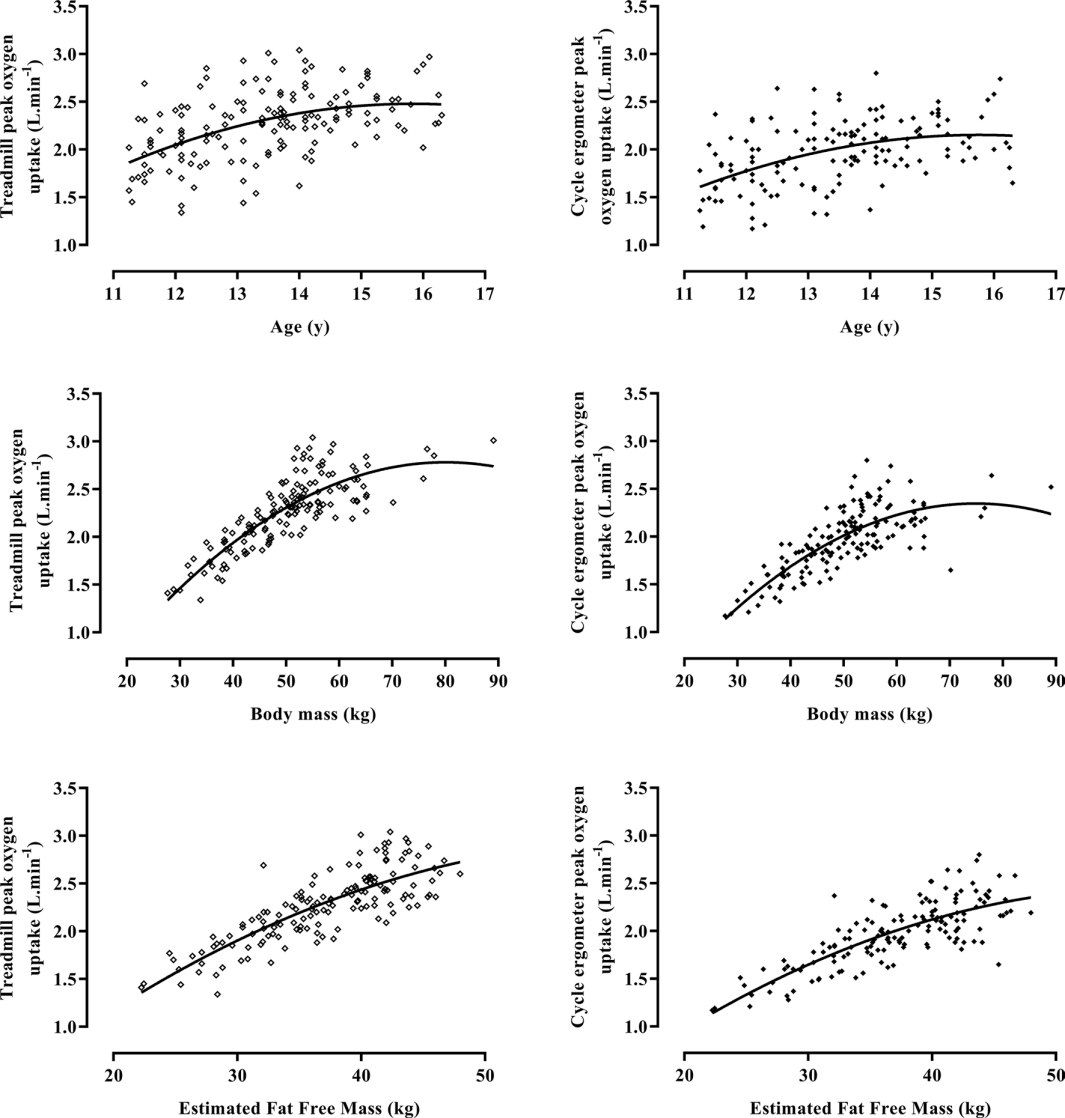
Treadmill and cycle ergometer-determined peak oxygen uptake by age, body mass, and fat-free mass in 11–16-year-old girls. Data are from 150 determinations of 11–16-year-old girls’ peak oxygen uptake on each ergometer. Fat-free mass is estimated from youth-specific equations (Slaughter et al. 1988)

Mean age-related treadmill-determined peak $\dot{{V}}{\text{O}}_{2}$ varied from 11 to 13% and 12 to 14% higher than cycle ergometer values in boys and girls, respectively, with the highest mean % difference being at age 13 years in both sexes. Ten boys and two girls recorded higher peak $\dot{{V}}{\text{O}}_{2}$ values on a cycle ergometer on one test occasion. Boys’ peak $\dot{{V}}{\text{O}}_{2}$ was significantly (*p* < 0.001) correlated with age (treadmill, *r* = 0.63; cycle ergometer, *r* = 0.64), body mass (treadmill, *r* = 0.86; cycle ergometer, *r* = 0.83), and estimated FFM (treadmill, *r* = 0.92; cycle ergometer, *r* = 0.92). Similarly, girls’ peak $\dot{{V}}{\text{O}}_{2}$ was significantly (*p* < 0.001) correlated with age (treadmill, *r* = 0.48; cycle ergometer, *r* = 0.46), body mass (treadmill, *r* = 0.79; cycle ergometer, *r* = 0.73), and estimated FFM (treadmill, *r* = 0.82; cycle ergometer, *r* = 0.79). The data are illustrated in Figs. 1 and 2.

### Multilevel models

Both the number of observations per individual and the temporal spacing of the observations may vary within a multilevel analysis enabling data representing 5 years to be collected over a 3 year period. In the present study, there were no significant differences (*p* > 0.05) between those who were unable to attend an annual test occasion and the rest of their age- and sex-specific group in body mass, skinfold thicknesses, or peak $\dot{{V}}{\text{O}}_{2}$. The analyses were founded on 640 (340 from boys) measurements of peak $\dot{{V}}{\text{O}}_{2}$ supported by age, body mass, skinfold thicknesses, estimated FFM and maturity status.

To explore sex differences in treadmill- and cycle ergometer-determined peak $\dot{{V}}{\text{O}}_{2}$, Table 1 includes the whole data set. Model 1.1 (treadmill) and Model 1.2 (cycle ergometer) confirm that with body mass controlled for there is an additional, significant positive effect of age on peak $\dot{{V}}{\text{O}}_{2}$. Both models show significant negative terms for sex and age by sex interaction with age^2^ terms not significant.

**Table 1:** Multilevel regression models for 11–16-year-old boys and girls combined for treadmill and cycle ergometer peak oxygen uptake

	Model 1.1 Log_e_ treadmill-determined peak oxygen uptake	Model 1.2 Log_e_ cycle ergometer-determined peak oxygen uptake
Parameters	Estimate (SE)	Estimate (SE)
Fixed part		
Constant	− 1.730 (0.150)	− 1.684 (0.166)
Log_e_ body mass	0.689 (0.039)	0.644 (0.043)
Age^a^	0.087 (0.014)	0.084 (0.015)
Age^2^	ns	ns
Sex	− 0.156 (0.016)	− 0.170 (0.018)
Age by sex	− 0.043 (0.008)	− 0.044 (0.009)
Random part		
Level 2		
Var(cons)	0.007 (0.001)	0.010 (0.001)
Level 1		
Var(cons)	0.004 (0.000)	0.004 (0.000)
Units: Level 2	136	136
Units: Level 1	320	320
− 2 × log-likelihood	− 635.011	− 607.099

Values are model estimates (standard error)

Table founded on 320 determinations of peak oxygen uptake on each ergometer

*ns* not significant (*p* > 0.05)

^a^Age centred on mean age 13.5 years

Tables 2 and 3 contain the models illustrating the boys’ treadmill and cycle ergometer data, respectively. Models 2.1 and 3.1 show both age and body mass to have significant, positive, effects on peak $\dot{{V}}{\text{O}}_{2}$ with age^2^ terms not significant in this or in other models. The entry of stages of PH development into Models 2.2 and 3.2, generated significant positive effects for PH stages 3–5 on peak $\dot{{V}}{\text{O}}_{2}$ in addition to those of body mass, with age becoming non-significant in both models. The introduction in Models 2.3 and 3.3 of sum of triceps and subscapular skinfold thicknesses generated negative exponents and resulted in large increases in the body mass exponents with age and maturity status becoming non-significant. Models 2.4 and 3.4 show no significant effects of age or maturity-status when estimated FFM replaced body mass, but the models based on estimated FFM were a significantly better statistical fit than those founded on body mass. A comparison of Models 2.3 and 3.3 with Models 2.4 with 3.4, respectively, for goodness of fit showed Models 2.3 and 3.3, with body mass and sum of skinfolds acting as a surrogate for FFM, to provide a better description of developmental changes in peak $\dot{{V}}{\text{O}}_{2}$ than Models 2.4 and 3.4 with estimated FFM as a covariate.

**Table 2:** Multilevel regression models for 11–16-year-old boys for treadmill-determined peak oxygen uptake

Parameter	Log_e_ treadmill-determined peak oxygen uptake			
	Model 2.1	Model 2.2	Model 2.3	Model 2.4
Fixed Part	Estimate (SE)	Estimate (SE)	Estimate (SE)	Estimate (SE)
Constant	− 1.900 (0.215)	− 1.774 (0.216)	− 2.287 (0.130)	− 2.330 (0.127)
Log_e_ Body mass	0.733 (0.056)	0.689 (0.057)	0.989 (0.035)	–
Age^a^	0.039 (0.009)	ns	ns	ns
Age^2^	ns	ns	ns	ns
Maturity stage 2	–	ns	ns	ns
Maturity stage 3	–	0.082 (0.022)	ns	ns
Maturity stage 4	–	0.061 (0.023)	ns	ns
Maturity stage 5	–	0.093 (0.031)	ns	ns
Log_e_ Skinfolds	–	–	− 0.207 (0.022)	–
Log_e_ FFM^b^	–	–	–	0.890 (0.034)
Random Part				
Level 2				
Var(cons)	0.009 (0.002)	0.008 (0.002)	0.005 (0.001)	0.006 (0.001)
Level 1				
Var(cons)	0.005 (0.001)	0.005 (0.001)	0.004 (0.001)	0.005 (0.001)
Units: Level 2	72	72	72	72
Units: Level 1	170	161	170	170
− 2 × log-likelihood	− 305.135	− 297.930	− 355.190	− 330.757

Values are model estimates (standard error)

Models 2.1, 2.3, and 2.4 founded on 170 determinations of peak oxygen uptake. Model 2.2 founded on 161 determinations of peak oxygen uptake

*ns* not significant (*p* > 0.05), – not entered

^a^Age centred on mean age 13.5 years

^b^Estimated fat free mass (Slaughter et al. 1988)

**Table 3:** Multilevel regression models for11-16 year-old boys for cycle ergometer-determined peak oxygen uptake

Parameter	Log_e_ cycle ergometer-determined peak oxygen uptake			
	Model 3.1	Model 3.2	Model 3.3	Model 3.4
Fixed Part	Estimate (SE)	Estimate (SE)	Estimate (SE)	Estimate (SE)
Constant	− 1.902 (0.228)	− 1.795 (0.209)	− 2.176 (0.128)	− 2.299 (0.117)
Log_e_ Body mass	0.700 (0.059)	0.660 (0.056)	0.936 (0.033)	–
Age^a^	0.034 (0.009)	ns	ns	ns
Age^2^	ns	ns	ns	ns
Maturity stage 2	–	ns	ns	ns
Maturity stage 3	–	0.081 (0.019)	ns	ns
Maturity stage 4	–	0.075 (0.019)	ns	ns
Maturity stage 5	–	0.111 (0.024)	ns	ns
Log_e_ Skinfolds	–	–	− 0.218 (0.021)	–
Log_e_ FFM^b^	–	–	–	0.847 (0.032)
Random Part				
Level 2				
Var(cons)	0.011 (0.002)	0.010 (0.002)	0.005 (0.001)	0.006 (0.001)
Level 1				
Var(cons)	0.004 (0.001)	0.004 (0.001)	0.003 (0.000)	0.004 (0.001)
Units: Level 2	72	72	72	72
Units: Level 1	170	161	170	170
− 2 × log-likelihood	− 311.897	− 302.871	− 374.066	− 359.525

Values are model estimates (standard error)

Models 3.1, 3.3, and 3.4 founded on 170 determinations of peak oxygen uptake. Model 3.2 founded on 161 determinations of peak oxygen uptake

*ns* not significant (*p* > 0.05), – not entered

^a^Age centred on mean age 13.5 years

^b^Estimated fat free mass (Slaughter et al. 1988)

The models describing the girls’ data are presented in Table 4. Models 4.1 (treadmill) and 4.4 (cycle ergometer) show the positive effect of body mass on peak $\dot{\text{V}}{\text{O}}_{2}.$ Age, age^2^, and maturity status demonstrated no additional, significant effects on peak $\dot{{V}}{\text{O}}_{2}$ in any of the models, as illustrated in Table 4. Models 4.2 and 4.5 show that the introduction of sum of skinfolds generated significant negative exponents coupled with increases in the size of the body mass exponents and no significant effect of age in either the treadmill or cycle ergometer models. A comparison revealed that Model 4.2, with body mass and sum of skinfolds acting as a surrogate for FFM, was a significantly better fit than Models 4.1 and 4.3, based on either body mass or estimated FFM, respectively. Both Model 4.5 and Model 4.6 were significantly better fits than Model 4.4.

**Table 4:** Multilevel regression models for 11–16-year-old girls for treadmill-determined and cycle ergometer-determined peak oxygen uptake

Parameter	Log_e_ treadmill-determined peak oxygen uptake			Log_e_ cycle ergometer-determined peak oxygen uptake		
	Model 4.1	Model 4.2	Model 4.3	Model 4.4	Model 4.5	Model 4.6
Fixed Part	Estimate (SE)	Estimate (SE)	Estimate (SE)	Estimate (SE)	Estimate (SE)	Estimate (SE)
Constant	− 1.776 (0.156)	− 1.808 (0.145)	− 2.011 (0.169)	− 1.562 (0.244)	− 1.699 (0.167)	− 1.974 (0.184)
Log_e_ Body mass	0.661 (0.040)	0.770 (0.043)	–	0.569 (0.062)	0.727 (0.050)	–
Age^a^	ns	ns	ns	ns	ns	ns
Age^2^	ns	ns	ns	ns	ns	ns
Maturity stage 2	ns	ns	ns	ns	ns	ns
Maturity stage 3	ns	ns	ns	ns	ns	ns
Maturity stage 4	ns	ns	ns	ns	ns	ns
Maturity stage 5	ns	ns	ns	ns	ns	ns
Log_e_ Skinfolds	–	− 0.121 (0.027)	–	–	− 0.148 (0.031)	–
Log_e_ FFM^b^	–	–	0.782 (0.047)	–	–	0.732 (0.051)
Random Part						
Level 2						
Var(cons)	0.005 (0.001)	0.004 (0.001)	0.005 (0.001)	0.008 (0.002)	0.006 (0.001)	0.007 (0.002)
Level 1						
Var(cons)	0.003 (0.000)	0.003 (0.000)	0.003 (0.000)	0.004 (0.001)	0.004 (0.001)	0.003 (0.001)
Units: Level 2	64	64	64	64	64	64
Units: Level 1	150	150	150	150	150	150
− 2 × log-likelihood	− 339.394	− 357.845	340.974	− 298.336	− 318.222	− 315.777

Values are model estimates (standard error)

All models founded on 150 determinations of peak oxygen uptake on each ergometer

*ns* not significant (*p* > 0.05), – not entered

^a^Age centred on mean age 13.5 years

^b^Estimated fat free mass (Slaughter et al. 1988)

## Discussion

The descriptive data and multilevel models demonstrate the need to distinguish between exercise modes and to adopt a sex-specific analysis of concurrent changes in anthropometric variables with age and maturity status when exploring the development of peak $\dot{{V}}{\text{O}}_{2}$ from 11–16 years. Multiplicative, multilevel models of peak $\dot{{V}}{\text{O}}_{2}$ were sex-specific but within sex models were similar on both ergometers. FFM was identified as the dominant morphological influence on the peak $\dot{{V}}{\text{O}}_{2}$ of both sexes.

Mean treadmill-determined peak $\dot{{V}}{\text{O}}_{2}$ values were higher than cycle ergometer-determined values at each test occasion but individual variations, with some children eliciting higher values on a cycle ergometer, illustrate the misjudgement of predicting treadmill-determined values by adding a fixed % regardless of age and sex. The individual data presented in Figs. 1 and 2 reflect previous cross-sectional (Armstrong and Welsman 1994) and longitudinal (Armstrong and McManus 2017) studies. On both ergometers peak $\dot{{V}}{\text{O}}_{2}$ increased with age, body mass, and estimated FFM but with wide individual variations, particularly in relation with age and less so in relation with estimated FFM. A statistical assumption underlying ratio scaling is a perfect correlation (i.e., *r* = 1.0) between peak $\dot{{V}}{\text{O}}_{2}$ and body mass (Tanner 1949; Katch 1973; Welsman and Armstrong 2018) which is showed not to be met in the current data set (*r* = ~ 0.73–0.86). The fallacy of the ratio-scaled peak $\dot{{V}}{\text{O}}_{2}$ interpretation of age-related aerobic fitness is reinforced by the allometric exponents identified for body mass in baseline models 1.1, 1.2, 2.1, 3.1, 4.1, and 4.4 ranging from 0.57 to 0.73 with an exponent of 1.0 (a necessary assumption in ratio scaling) falling outside the 95% confidence limits on each occasion. Descriptions of youth peak $\dot{{V}}{\text{O}}_{2}$ in ratio with single anthropometric variables are ‘convenient and traditional’ (Bar-Or and Rowland 2004), but both the descriptive data and all models are in direct conflict with the ratio-scaled interpretation of youth peak $\dot{{V}}{\text{O}}_{2}$ (Welsman and Armstrong 2018). Collectively, they expose the fallacy of using peak $\dot{{V}}{\text{O}}_{2}$ values in ratio with body mass as cut-points for healthy levels of aerobic fitness or for raising ‘clinical red flags’ for boys and girls between 11 and 16 years of age.

The baseline models 1.1 and 1.2 illustrated in Table 1 are each founded on 320 peak $\dot{{V}}{\text{O}}_{2}$ determinations and they illustrate that data from both ergometers present a similar picture of the development of peak $\dot{{V}}{\text{O}}_{2}$ from 11 to 16 years of age. The models demonstrate that with body mass controlled for both treadmill- and cycle ergometer-determined peak $\dot{{V}}{\text{O}}_{2}$s increase with age in both sexes. The negative sex term shows boys’ peak $\dot{{V}}{\text{O}}_{2}$ to be higher than girls’ values and the negative age by sex interaction term demonstrates that peak $\dot{{V}}{\text{O}}_{2}$ increases with age at a greater rate in boys. Sex differences have generally been attributed to lower maximal SVs in girls (Rowland et al. 2000; Vinet et al. 2003), although girls have also been reported to have significantly lower maximal arterio-venous oxygen differences (a-vO_2_ diff max) (Winsley et al. 2009) and poorer matching of muscle oxygen delivery to oxygen utilization (McNarry et al. 2015). However, although the physiological mechanisms underlying sex differences in youth aerobic fitness are emerging they remain to be fully elucidated (for review, see Armstrong and McManus 2017).

Girls normally enter puberty before similarly aged boys and, for example, a 13-year-old girl in PH stage 4 is not equivalent to a 15-year-old boy at the same pubertal stage (Malina 2017). It is, therefore, appropriate to analyse data in sex-specific models (Tables 2, 3, 4) which provide more sensitive explorations of the development of girls’ and boys’ peak $\dot{{V}}{\text{O}}_{2}$ than the combined data in Table 1. The within sex models of treadmill- and cycle ergometer-determined peak $\dot{{V}}{\text{O}}_{2}$ in Tables 2 and 3 (boys) and Table 4 (girls) are remarkably similar and suggest that, although the magnitude of peak $\dot{{V}}{\text{O}}_{2}$ is exercise mode-specific, either ergometer can be used to interpret the development of boys’ or girls’ aerobic fitness. Although there were marked sex differences in the relative concurrent contributions of age, body mass, and maturity status, FFM was the most powerful influence on peak $\dot{{V}}{\text{O}}_{2}$ in both sexes.

In boys, baseline models 2.1 and 3.1 show that age exerts a positive effect on peak $\dot{{V}}{\text{O}}_{2}$ in addition to body mass. When maturity status was entered, as in Models 2.2 and 3.2, PH stages 3–5 were shown to exert significant, positive effects on both treadmill- and cycle ergometer-determined peak $\dot{{V}}{\text{O}}_{2}$ in addition to those of body mass, with the age exponent becoming non-significant. The additional effect of maturity status on peak $\dot{{V}}{\text{O}}_{2}$ not only reflects the development of cardiorespiratory variables but also factors such as increasing muscle mass and, therefore, FFM. The introduction of sum of skinfold thicknesses into Models 2.3 and 3.3 provided the best statistical fit for both treadmill and cycle ergometer data with significant negative exponents for sum of skinfolds and increased body mass exponents. The marked increase in the body mass exponents with the introduction of skinfold thicknesses has been previously observed and attributed to the effect that excess fat mass has on increasing body mass without an increase in the exercise variable (Vanderburgh et al. 1995). The models founded on estimated FFM (i.e., Models 2.4 and 3.4) were also superior to models based on body mass (i.e., Models 2.1 and 3.1). Taken together, skinfold thicknesses and body mass provide a surrogate for FFM which explains more of the variance in peak $\dot{{V}}{\text{O}}_{2}$ than the estimation of FFM from the youth-specific equations developed by Slaughter et al. (1988). The introduction of body mass and skinfold thicknesses (or estimated FFM) masks the effects of age and maturity status and demonstrates the strong influence of FFM on peak $\dot{{V}}{\text{O}}_{2}$.

The girls’ models 4.1 and 4.4 for treadmill and cycle ergometer data, respectively, revealed a different story as the baseline models were not statistically improved by the introduction of stages of maturation. This finding is in agreement with Nevill et al.’s (1998) seminal multilevel modelling study where in boys, but not girls, maturity status effects on the peak $\dot{{V}}{\text{O}}_{2}$ of elite athletes were additional to those due to body size. In contrast, our multilevel modelling study over a longer age range (10–18 years) and founded on 1057 treadmill determinations of peak $\dot{{V}}{\text{O}}_{2}$ noted maturity status to have significant effects in addition to those of age and body mass in both sexes (Armstrong and Welsman 2018c). Nevertheless, as with boys, the strongest statistical model (i.e., Model 4.2) for treadmill-determined peak $\dot{{V}}{\text{O}}_{2}$ was created by the introduction of sum of skinfolds which resulted in a negative skinfold exponent and a concurrent increase in the body mass exponent. Model 4.5, with body mass and skinfold thicknesses acting as a surrogate of FFM, and Model 4.6, founded on estimated FFM, were the strongest statistical models of cycle ergometer data. These findings emphasise the dominant effect of changes in FFM on the development of peak $\dot{{V}}{\text{O}}_{2}$ in both sexes on both ergometers.

Increases in peak $\dot{{V}}{\text{O}}_{2}$ are manifest through changes in SVmax, a-vO_2_ diff max, or both but increases in muscle mass, reflected by gains in FFM, not only enhance total muscle $\dot{{V}}{\text{O}}_{2}$ during exercise but, through the peripheral muscle pump, also augment venous return to the heart and increase SVmax. Driven by maturation, FFM increases by ~ 40% and ~ 90% in girls and boys, respectively, from 11 to 16 years. The influence of the timing and tempo of maturation on FFM is evidenced in boys by an ~ 83% increase over the period 2 years pre-peak height velocity (PHV) to 2 years post-PHV. The greatest increase in girls’ FFM (~ 31%) occurs over a shorter 2-year period centred on PHV and then levels-off in accord with the development of peak $\dot{{V}}{\text{O}}_{2}$ (Armstrong 2018)

In conclusion, the present data demonstrate that (1) changes in maturity status-driven FFM exert a powerful influence on the development of peak $\dot{\text{V}}{\text{O}}_{2}$from 11 to 16 years, in both sexes on both ergometers; (2) the use of concurrent changes in body mass and skinfold thicknesses as a surrogate for FFM can be recommended for future investigations of the development of peak $\dot{{V}}{\text{O}}_{2}$; (3) it is untenable to base interpretation of youth aerobic fitness on peak $\dot{{V}}{\text{O}}_{2}$ ratio-scaled with body mass; (4) it is misleading to combine treadmill and cycle ergometer data or to use fixed conversion factors to ‘correct’ for differences when investigating the development of peak $\dot{{V}}{\text{O}}_{2}$; and (5) current ‘clinical red flags’ (and similar health-related peak $\dot{{V}}{\text{O}}_{2}$ cut-points) established without consideration of exercise mode and founded on peak $\dot{{V}}{\text{O}}_{2}$ ratio-scaled with body mass are fallacious and have the potential to misinform clinical practice and misguide recommendations designed to promote youth health.

## Abbreviations

a-vO_2_ diff: Arterio-venous oxygen difference
FFM: Fat-free mass
HR: Heart rate
$$\dot{{V}}{\text{O}}_{2}$$: Oxygen uptake
PHV: Peak height velocity
PH: Pubic hair
SV: Stroke volume

## References

[CR1] Aadland E, Anderssen SA, Andersen LB, Resaland GK, Kolle E, Johannessen (2018). Aerobic thresholds to define poor metabolic health in children. Scand J Med Sci Sports.

[CR2] Agbaje AO, Haapala EA, Lintu N (2018). Peak oxygen uptake cut points to identify children at increased cardiometabolic risk—the PANIC Study. Scand J Med Sci.

[CR3] Aitkin M, Anderson D, Hinde J (1981). Statistical modelling of data on teaching styles. J Roy Stat Soc A.

[CR4] Armstrong N (2018). Development of the youth athlete.

[CR5] Armstrong N, McManus AM, Armstrong N, van Mechelen W (2017). Aerobic fitness. Oxford textbook of children’s sport and exercise medicine.

[CR6] Armstrong N, Welsman JR (1994). Assessment and interpretation of aerobic fitness in children and adolescents. Exerc Sport Sci Rev.

[CR7] Armstrong N, Welsman J (2018). Sex-specific longitudinal modelling of short-term power in 11–18 year-olds. Med Sci Sport Exerc.

[CR8] Armstrong N, Welsman J (2018). Editorial: The 20-m shuttle run: (mis)representation, (mis)interpretation, and (mis)use. Br J Sports Med.

[CR9] Armstrong N, Welsman J (2018). Sex-specific longitudinal modelling of youth aerobic fitness. Pediatr Exerc Sci.

[CR10] Armstrong N, Williams J, Balding J, Gentle P, Kirby B (1991). Peak oxygen uptake of British children with reference to age, sex and sexual maturity. Eur J Appl Physiol.

[CR11] Armstrong N, Welsman JR, Nevill AM, Kirby BJ (1999). Modeling growth and maturation changes in peak oxygen uptake in 11–13 year olds. J Appl Physiol.

[CR12] Bacquet G, Van Praagh E, Berthoin S (2003). Endurance training and endurance fitness in young people. Sports Med.

[CR13] Bar-Or O, Rowland TW (2004). Pediatric exercise medicine.

[CR14] Boileau RA, Bonen A, Heyward VH, Massey BH (1977). Maximal aerobic capacity on the treadmill and bicycle ergometer of boys 11–14 years of age. J Sports Med Phys Fit.

[CR15] Cunningham DA, Paterson DH, Blimkie CJR, Donner AP (1994). Development of cardiorespiratory function in circumpubertal boys: a longitudinal study. J Appl Physiol.

[CR16] De Ste Croix MBA, Armstrong N, Welsman JR, Sharpe P (2002). Longitudinal changes in isokinetic leg strength in 1014-year-olds. Ann Hum Biol.

[CR17] Duncan GE, Mahon AD, Gay JA, Sherwood JJ (1996). Physiological and perceptual responses to graded treadmill and cycle exercise in male children. Pediatr Exerc Sci.

[CR18] Falk B, Dotan R (2018). Measurement and interpretation of maximal aerobic power in children. Pediatr Exerc Sci doi.

[CR19] Ferri-Morales A, Nascimento-Ferreira MV, Vlachopoulos D (2018). Agreement between standard body composition methods to estimate percentage of body fat in young male athletes. Pediatr Exerc Sci.

[CR20] Goran M, Fields DA, Hunter GR, Herd SL, Weinster RL (2000). Total body fat does not influence maximal aerobic capacity. Int J Obes.

[CR21] Janz KF, Burns TL, Witt JD, Mahoney LT (1998). Longitudinal analysis of scaling for differences in body size during puberty: the Muscatine study. Med Sci Sports Exerc.

[CR22] Katch VL (1973). Use of the oxygen/body weight ratio in correlational analyses: spurious relationships and statistical considerations. Med Sci Sports.

[CR24] Krahenbuhl GS, Skinner JS, Kohrt WM (1985). Developmental aspects of maximal aerobic power in children. Exerc Sports Sci Rev.

[CR25] Lang JJ, Tremblay MS, Ortega FB, Ruiz JR, Tomkinson GR (2017). Review of criterion-referenced standards for cardiorespiratory fitness: what percentage of 1142026 international children and youth are apparently healthy?. Br J Sports Med.

[CR26] Loftin M, Sothern M, Abe T, Bonis M (2016). Expression of _peak_ in children and youth with special reference to allometric scaling. Sports Med.

[CR27] Macek M, Vavra J, Novosadova J (1976). Prolonged exercise in prepubertal boys. Eur J Appl Physiol.

[CR28] Malina RM, van ArmstrongMechelen NW (2017). Assessment of maturation. Oxford textbook of children’s sport and exercise medicine.

[CR29] Mamen A, Resaland GK, Mo DA, Andersen LB, Jürimäe T, Armstrong N, Jürimäe J (2009). Comparison of peak oxygen uptake in boys exercising on treadmill and cycle ergometers. Children and exercise XXIV.

[CR30] McManus AM, Armstrong N, Rowland TW (2017). Maximal oxygen uptake. Cardiopulmonary exercise testing in children and adolescents.

[CR31] McNarry MA, Farr C, Middlebrooke A, Welford D, Breese B, Armstrong N, Barker AR (2015). Aerobic function and muscle deoxygenation dynamics during ramp exercise in children. Med Sci Sports Exerc.

[CR32] Mirwald RL, Bailey DA (1986). Maximal aerobic power.

[CR33] Nevill AM, Holder RL, Baxter-Jones A, Round JM, Jones DA (1998). Modelling developmental changes in strength and aerobic power in children. J Appl Physiol.

[CR34] Pfeiffer K, Lobelo F, Ward DS, Pate RR, Hebestreit H, Bar-Or O (2008). Endurance trainability of children and youth. The young athlete.

[CR35] Rasbash J, Steele F, Browne WJ, Goldstein H (2018). A user’s guide to MLwiN Version 3.02.

[CR36] Roemmich JN, Clark PA, Weltman A, Rogol AD (1997). Alterations in growth and body composition during puberty. I. Comparing multicompartment body composition models. J Appl Physiol.

[CR37] Rowland T, Vanderburgh P, Cunningham L (1997). Body size and the growth of maximal aerobic power in children: a longitudinal analysis. Pediatr Exerc Sci.

[CR38] Rowland T, Goff D, Martel L, Ferrone L (2000). Influence of cardiac functional capacity on gender differences in maximal oxygen uptake in children. Chest.

[CR39] Ruiz JR, Cavero-Redondo I, Ortega FB, Welk GJ, Andersen LB, Martinez-Vizcaino V (2016). Cardiorespiratory fitness cut points to avoid cardiovascular disease risk in children and adolescents: what level of fitness should raise a red flag? A systematic review and meta-analysis. Br J Sports Med.

[CR40] Rutenfranz J, Andersen KL, Seliger V, Klimmer F, Berndt I, Ruppel M (1981). Maximum aerobic power and body composition during the puberty growth period: similarities and differences between children of two European countries. Eur J Pediatr.

[CR41] Slaughter MH, Lohman TG, Boileau RA, Horswill CA, Stillman RJ, Van Loan MD, Bemben DA (1988). Skinfold equations for estimation of body fatness in children and youth. Hum Biol.

[CR42] Sprynarova S, Parizkova J, Bunc S (1987). Relationships between body dimensions and resting and working oxygen consumption in boys aged 11 to 18 years. Eur J Appl Physiol.

[CR43] Stavnsbo M, Resaland GK, Anderssen SA, Steene-Johannessen J, Domazet SL, Skrede T, Sardinha LB, Kriemler S, Ekelund U, Andersen LB, Aadland E (2018). Reference values for cardiometabolic risk scores in children and adolescents: Suggesting a common standard. Atherosclerosis.

[CR44] Tanner JM (1949). Fallacy of per-weight and per-surface area standards and their relation to spurious correlation. J Appl Physiol.

[CR45] Tanner JM (1962). Growth at adolescence.

[CR46] Vanderburgh PM, Mahar MT, Chou CH (1995). Allometric scaling of grip strength by body mass in college-age men and women. Res Q Exerc Sport.

[CR47] Vinet A, Mandigout S, Nottin S, Nguyen LD, Lecoq A-M, Courteix D, Obert P (2003). Influence of body composition, hemoglobin concentration, and cardiac size and function on gender differences in maximal oxygen uptake in prepubertal children. Chest.

[CR48] Welsman J, Armstrong N (2018). Interpreting ratio scaling in youth: the fallacy of ratio scaling. Pediatr Exerc Sci.

[CR49] Welsman JR, Armstrong N, Kirby BJ, Nevill AM, Winter EM (1996). Scaling peak for differences in body size. Med Sci Sports Exerc.

[CR50] Winsley RJ, Fulford J, Roberts AC, Welsman JR, Armstrong N (2009). Sex difference in peak oxygen uptake in prepubertal children. J Sci Med Sport.

[CR51] Winter EW, Nevill AM, Eston R, Reilly T (2009). Scaling: Adjusting for differences in body size. Kinanthropometry and exercise physiology laboratory manual.

